# *C*-type Yb_2_Te_3_O_9_

**DOI:** 10.1107/S2414314624008885

**Published:** 2024-09-17

**Authors:** Patrick Höss, Sheng-Chun Chou, Philip L. Russ, Ralf J. C. Locke, Thomas Schleid

**Affiliations:** a Institut für Anorganische Chemie, Universität Stuttgart, Pfaffenwaldring, 55, 70569 Stuttgart, Germany; Vienna University of Technology, Austria

**Keywords:** crystal structure, oxidotellurates(IV), lanthanoids, ytterbium

## Abstract

The title compound, diytterbium enneaoxidotritellurate(IV), was obtained in its *C*-type crystal structure from the binary oxides at 1073 K using a CsCl flux. It crystallizes isotypically with *C*-type Tm_2_Te_3_O_9_ and Lu_2_Te_3_O_9_, closing this gap of knowledge.

## Abstract 2

Yb_2_Te_3_O_9_ is isotypic with Tm_2_Te_3_O_9_ and Lu_2_Te_3_O_9_ and crystallizes in the *C*-type modification of *Ln*_2_Te_3_O_9_ compounds.

## Graphical abstract

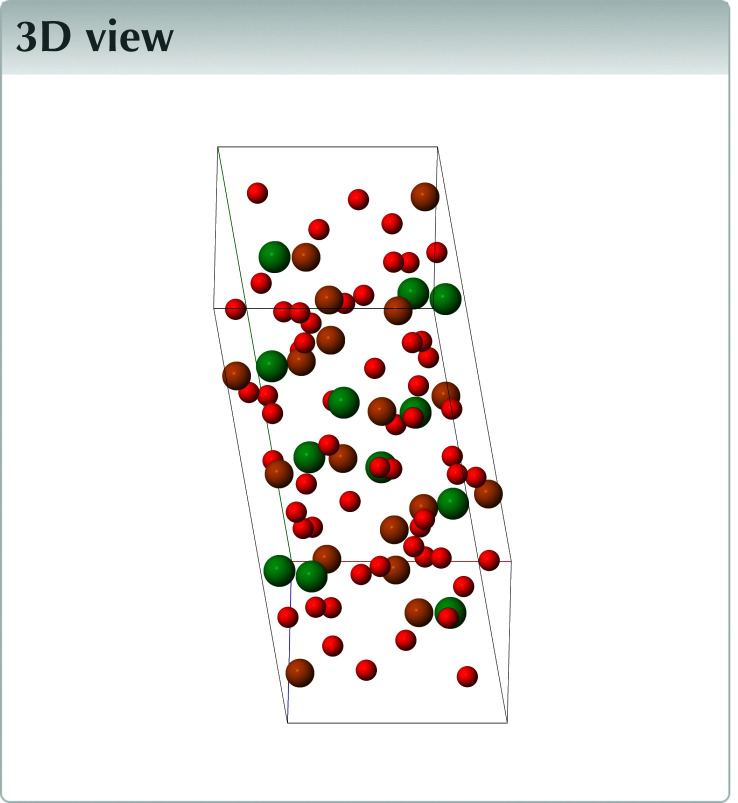

## Structure description

The lanthanoid(III) oxidotellurates(IV) of the formula type *Ln*_2_Te_3_O_9_ exhibit three structure types depending on the size of the involved *Ln*^3+^ cation. With the monoclinic *A*1 type for *Ln* = La and Ce and the highly related *A*2 type for *Ln* = Pr and Nd (Chou *et al.*, 2021 ▸), the likewise monoclinic Dy_2_Te_3_O_9_ in the *B* type (Meier *et al.*, 2009 ▸) and the triclinic *C* type for *Ln* = Tm (Höss & Schleid, 2008 ▸) and Lu (Höss *et al.*, 2013 ▸), all these structure types could be characterized on the basis of single-crystal X-ray diffraction data.

**Figure 1:**
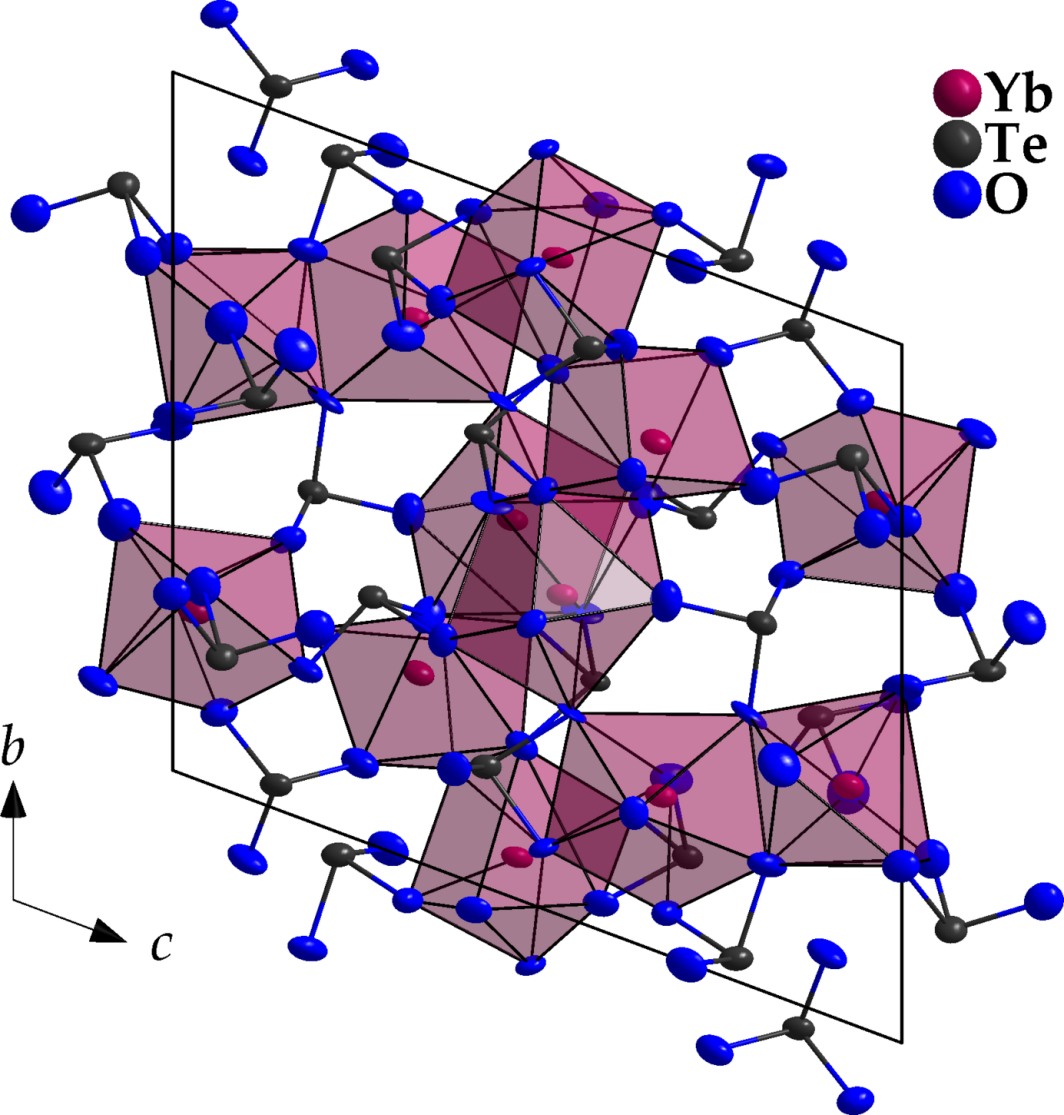
The triclinic crystal structure of *C*-type Yb_2_Te_3_O_9_ in a view along [100]. Displacement ellipsoids are drawn at the 95% probability level.

Yb_2_Te_3_O_9_ crystallizes isotypically with Tm_2_Te_3_O_9_ and Lu_2_Te_3_O_9_ in the triclinic space group *P*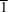. All atoms in the crystal structure occupy the general position 2*i*. The six crystallographically different Yb^3+^ cations are surrounded seven- or eightfold by oxygen [*d*(Yb—O) = 2.130 (6)–2.737 (6) Å] and the resulting distorted coordination polyhedra undergo condensation *via* common corners and edges to form [Yb_6_O_27_]^36–^ layers with a pronounced profile structure parallel to (001). The nine distinct tellurium atoms are each surrounded in the primary coordination sphere by three oxygen atoms with typical distances *d*(Te—O) = 1.844 (7)–1.955 (6) Å, which together with the free, non-bonding electron pairs of the Te^4+^ cations result in a ψ^1^-tetra­hedral shape of the corresponding oxidotellurate(IV) anions. In addition, secondary inter­actions typical for lanthanoid(III) oxidotellurates(IV) also occur between several [TeO_3_]^2–^ units [*d*(Te⋯O) = 2.380 (6)-2.729 (7) Å]. So two of the [TeO_3_]^2–^ anions link the [Yb_6_O_27_]^36–^ layers in the [001] direction, resulting in a tri-periodic network. This leaves sufficient space between the layers for the free non-bonding electron pairs of the Te^4+^ cations (Fig. 1 ▸).

## Synthesis and crystallization

Based on the general formula (Yb_2_O_3_)(TeO_2_)_*n*_, the corresponding oxides Yb_2_O_3_ and TeO_2_ in a molar ratio of 1:3 and an excess of caesium chloride, CsCl, as flux were reacted in evacuated silica glass ampoules at 1073 K for 10 d for this specific preparation with *n* = 3. In that way, hydrolysis- and air-resistant single crystals of Yb_2_Te_3_O_9_ were obtained in the form of colourless and transparent crystals.

## Refinement

Crystal data, data collection and structure refinement details are summarized in Table 1 ▸. For the structure solution, coordinates were taken from an isotypic compound.

## Supplementary Material

Crystal structure: contains datablock(s) I. DOI: 10.1107/S2414314624008885/wm4219sup1.cif

Structure factors: contains datablock(s) I. DOI: 10.1107/S2414314624008885/wm4219Isup2.hkl

CCDC reference: 2383284

Additional supporting information:  crystallographic information; 3D view; checkCIF report

## Figures and Tables

**Table 1:** Experimental details

Crystal data	
Chemical formula	O_9_Te_3_Yb_2_
*M*_r_	872.88
Crystal system, space group	Triclinic, *P* 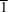
Temperature (K)	293
*a*, *b*, *c* (Å)	6.9208 (4), 13.2576 (8), 14.5513 (9)
α, β, γ (°)	110.068 (3), 90.497 (3), 100.082 (3)
*V* (Å^3^)	1231.29 (13)
*Z*	6
Radiation type	Mo *K*α
μ (mm^−1^)	33.12
Crystal size (mm)	0.05 × 0.04 × 0.02

Data collection	
Diffractometer	Nonius Kappa-CCD
Absorption correction	Numerical [*X-SHAPE* (Stoe, 2020 ▸) based on *HABITUS* (Herrendorf, 1995 ▸)]
*T*_min_, *T*_max_	0.107, 0.129
No. of measured, independent and observed [*I* > 2σ(*I*)] reflections	43221, 6120, 5334
*R*_int_	0.099
(sin θ/λ)_max_ (Å^−1^)	0.667

Refinement	
*R*[*F*^2^ > 2σ(*F*^2^)], *wR*(*F*^2^), *S*	0.033, 0.065, 1.06
No. of reflections	6120
No. of parameters	375
Δρ_max_, Δρ_min_ (e Å^−3^)	2.05, −2.15

Computer programs: *COLLECT* (Nonius, 1998 ▸), *DENZO*/*SCALEPACK* (Otwinowski & Minor, 1997 ▸), *SHELXL* (Sheldrick, 2015 ▸), *DIAMOND* (Brandenburg & Putz, 2005 ▸) and *publCIF* (Westrip, 2010 ▸).

## full crystallographic data

### Diytterbium enneaoxidotritellurate(IV) Crystal data

**Table:**

O_9_Te_3_Yb_2_	*Z* = 6
*M**_r_* = 872.88	*F*(000) = 2208
Triclinic, *P*1	*D*_x_ = 7.063 Mg m^−^^3^
*a* = 6.9208 (4) Å	Mo *K*α radiation, λ = 0.71069 Å
*b* = 13.2576 (8) Å	Cell parameters from 5334 reflections
*c* = 14.5513 (9) Å	θ = 0.4–28.3°
α = 110.068 (3)°	µ = 33.12 mm^−^^1^
β = 90.497 (3)°	*T* = 293 K
γ = 100.082 (3)°	Bar, colourless
*V* = 1231.29 (13) Å^3^	0.05 × 0.04 × 0.02 mm

### Diytterbium enneaoxidotritellurate(IV) Data collection

**Table:**

Nonius Kappa-CCD diffractometer	5334 reflections with *I* > 2σ(*I*)
Radiation source: fine-focus sealed tube	*R*_int_ = 0.099
four–circle, CCD–detector scans	θ_max_ = 28.3°, θ_min_ = 1.5°
Absorption correction: numerical [X-SHAPE (Stoe, 2020) based on HABITUS (Herrendorf, 1995)]	*h* = −9→9
*T*_min_ = 0.107, *T*_max_ = 0.129	*k* = −17→17
43221 measured reflections	*l* = −19→19
6120 independent reflections	

### Diytterbium enneaoxidotritellurate(IV) Refinement

**Table:**

Refinement on *F*^2^	0 restraints
Least-squares matrix: full	*w* = 1/[σ^2^(*F*_o_^2^) + (0.0222*P*)^2^ + 9.0186*P*] where *P* = (*F*_o_^2^ + 2*F*_c_^2^)/3
*R*[*F*^2^ > 2σ(*F*^2^)] = 0.033	(Δ/σ)_max_ = 0.001
*wR*(*F*^2^) = 0.065	Δρ_max_ = 2.05 e Å^−^^3^
*S* = 1.06	Δρ_min_ = −2.15 e Å^−^^3^
6120 reflections	Extinction correction: SHELXL (Sheldrick, 2015), Fc^*^=kFc[1+0.001xFc^2^λ^3^/sin(2θ)]^-1/4^
375 parameters	Extinction coefficient: 0.00102 (3)

### Diytterbium enneaoxidotritellurate(IV) Special details

**Table:**

Geometry. All esds (except the esd in the dihedral angle between two l.s. planes) are estimated using the full covariance matrix. The cell esds are taken into account individually in the estimation of esds in distances, angles and torsion angles; correlations between esds in cell parameters are only used when they are defined by crystal symmetry. An approximate (isotropic) treatment of cell esds is used for estimating esds involving l.s. planes.

### Diytterbium enneaoxidotritellurate(IV) Fractional atomic coordinates and isotropic or equivalent isotropic displacement parameters (Å^2^)

**Table:**

	*x*	*y*	*z*	*U*_iso_*/*U*_eq_	
Yb1	0.24579 (5)	0.45962 (3)	0.53421 (3)	0.00751 (9)	
Yb2	0.75209 (5)	0.05988 (3)	0.47217 (3)	0.00807 (9)	
Yb3	0.83241 (5)	0.27086 (3)	0.33711 (3)	0.00854 (9)	
Yb4	0.17952 (5)	0.22483 (3)	0.66972 (3)	0.00771 (9)	
Yb5	0.07557 (6)	0.33879 (3)	0.92671 (3)	0.00988 (9)	
Yb6	0.48911 (5)	0.23859 (3)	0.02980 (3)	0.00880 (9)	
Te1	0.22726 (8)	0.17280 (4)	0.42919 (4)	0.00798 (12)	
Te2	0.35619 (8)	0.84828 (4)	0.29392 (4)	0.00790 (12)	
Te3	0.06320 (8)	0.03237 (5)	0.77302 (5)	0.00941 (12)	
Te4	0.35869 (8)	0.35524 (4)	0.27413 (4)	0.00725 (12)	
Te5	0.26918 (8)	0.64602 (4)	0.42041 (4)	0.00723 (12)	
Te6	0.96044 (8)	0.18865 (5)	0.06765 (5)	0.00953 (12)	
Te7	0.48888 (8)	0.03448 (4)	0.14179 (4)	0.00901 (12)	
Te8	0.86476 (8)	0.47376 (4)	0.19231 (4)	0.00831 (12)	
Te9	0.37548 (9)	0.57923 (5)	0.11773 (5)	0.01079 (13)	
O1	0.1500 (9)	0.0878 (5)	0.5091 (5)	0.0093 (13)	
O2	0.9580 (9)	0.1526 (5)	0.3857 (5)	0.0109 (13)*	
O3	0.2053 (9)	0.2959 (4)	0.5478 (5)	0.0092 (13)	
O4	0.4553 (9)	0.9614 (5)	0.4129 (5)	0.0102 (13)	
O5	0.4979 (9)	0.7456 (5)	0.3160 (5)	0.0123 (14)	
O6	0.1423 (8)	0.8142 (5)	0.3675 (5)	0.0094 (13)	
O7	0.2210 (9)	0.0602 (5)	0.6778 (5)	0.0114 (13)	
O8	0.1877 (9)	0.0034 (5)	0.2954 (5)	0.0139 (14)	
O9	0.0609 (9)	0.1830 (5)	0.8150 (5)	0.0134 (14)	
O10	0.3427 (9)	0.2150 (5)	0.1816 (5)	0.0129 (13)	
O11	0.5833 (9)	0.3595 (5)	0.3504 (5)	0.0112 (13)	
O12	0.1857 (8)	0.3312 (5)	0.3706 (5)	0.0094 (13)	
O13	0.6398 (9)	0.2220 (5)	0.4785 (5)	0.0109 (13)	
O14	0.4378 (9)	0.5542 (5)	0.4382 (5)	0.0114 (13)	
O15	0.8990 (8)	0.4020 (5)	0.4921 (5)	0.0079 (12)	
O16	0.9367 (9)	0.2788 (5)	0.1950 (5)	0.0155 (14)	
O17	0.1832 (9)	0.7473 (5)	0.0014 (5)	0.0111 (13)	
O18	0.1860 (9)	0.2794 (5)	0.0409 (5)	0.0128 (14)	
O19	0.6493 (10)	0.1106 (5)	0.2568 (5)	0.0146 (14)	
O20	0.3866 (10)	0.0876 (5)	0.8976 (5)	0.0161 (14)	
O21	0.6237 (9)	0.1077 (5)	0.0636 (5)	0.0127 (13)	
O22	0.9011 (10)	0.4933 (5)	0.3234 (5)	0.0164 (14)	
O23	0.6099 (9)	0.3913 (5)	0.1583 (5)	0.0111 (13)	
O24	0.1981 (9)	0.3877 (5)	0.7905 (5)	0.0111 (13)	
O25	0.4447 (10)	0.3299 (6)	0.9266 (6)	0.0213 (16)	
O26	0.1925 (10)	0.6675 (6)	0.1681 (5)	0.0196 (15)	
O27	0.2116 (14)	0.5093 (6)	0.0019 (6)	0.037 (2)	

### Diytterbium enneaoxidotritellurate(IV) Atomic displacement parameters (Å^2^)

**Table:**

	*U*^11^	*U*^22^	*U*^33^	*U*^12^	*U*^13^	*U*^23^
Yb1	0.00545 (17)	0.00573 (17)	0.0114 (2)	0.00094 (14)	0.00023 (15)	0.00313 (15)
Yb2	0.00707 (18)	0.00507 (17)	0.0115 (2)	0.00071 (14)	0.00108 (15)	0.00243 (15)
Yb3	0.00709 (18)	0.00659 (17)	0.01136 (19)	0.00226 (14)	−0.00040 (15)	0.00197 (15)
Yb4	0.00500 (17)	0.00603 (17)	0.01166 (19)	0.00071 (14)	−0.00043 (15)	0.00274 (15)
Yb5	0.01007 (18)	0.00722 (18)	0.0111 (2)	−0.00072 (14)	−0.00103 (15)	0.00284 (15)
Yb6	0.00642 (18)	0.00662 (17)	0.0129 (2)	0.00124 (14)	−0.00043 (15)	0.00286 (15)
Te1	0.0054 (3)	0.0072 (3)	0.0115 (3)	0.0005 (2)	0.0001 (2)	0.0037 (2)
Te2	0.0058 (3)	0.0068 (3)	0.0109 (3)	0.0012 (2)	0.0008 (2)	0.0027 (2)
Te3	0.0080 (3)	0.0078 (3)	0.0132 (3)	0.0014 (2)	0.0010 (2)	0.0048 (2)
Te4	0.0058 (3)	0.0065 (3)	0.0098 (3)	0.0018 (2)	0.0001 (2)	0.0030 (2)
Te5	0.0064 (3)	0.0048 (2)	0.0103 (3)	0.0008 (2)	0.0000 (2)	0.0025 (2)
Te6	0.0085 (3)	0.0091 (3)	0.0123 (3)	0.0038 (2)	0.0017 (2)	0.0043 (2)
Te7	0.0078 (3)	0.0066 (3)	0.0121 (3)	−0.0004 (2)	−0.0010 (2)	0.0036 (2)
Te8	0.0070 (3)	0.0063 (3)	0.0108 (3)	0.0009 (2)	0.0005 (2)	0.0021 (2)
Te9	0.0107 (3)	0.0075 (3)	0.0156 (3)	0.0023 (2)	0.0016 (2)	0.0057 (2)
O1	0.011 (3)	0.007 (3)	0.011 (3)	0.002 (2)	0.001 (3)	0.006 (3)
O3	0.015 (3)	0.002 (3)	0.009 (3)	0.003 (2)	0.001 (3)	−0.001 (2)
O4	0.006 (3)	0.008 (3)	0.015 (3)	−0.002 (2)	−0.002 (3)	0.004 (3)
O5	0.006 (3)	0.012 (3)	0.024 (4)	0.002 (2)	0.004 (3)	0.011 (3)
O6	0.004 (3)	0.012 (3)	0.010 (3)	−0.001 (2)	0.000 (2)	0.003 (3)
O7	0.015 (3)	0.008 (3)	0.012 (3)	0.002 (2)	0.003 (3)	0.004 (3)
O8	0.007 (3)	0.012 (3)	0.020 (4)	−0.001 (2)	−0.004 (3)	0.003 (3)
O9	0.014 (3)	0.007 (3)	0.018 (4)	0.000 (2)	0.005 (3)	0.004 (3)
O10	0.017 (3)	0.006 (3)	0.014 (3)	0.001 (3)	−0.006 (3)	0.001 (3)
O11	0.007 (3)	0.017 (3)	0.014 (3)	0.007 (3)	0.002 (3)	0.009 (3)
O12	0.004 (3)	0.014 (3)	0.010 (3)	−0.002 (2)	−0.001 (2)	0.005 (3)
O13	0.011 (3)	0.008 (3)	0.012 (3)	0.001 (2)	−0.001 (3)	0.001 (3)
O14	0.007 (3)	0.006 (3)	0.022 (4)	0.005 (2)	0.001 (3)	0.004 (3)
O15	0.005 (3)	0.010 (3)	0.010 (3)	0.001 (2)	−0.001 (2)	0.006 (3)
O16	0.011 (3)	0.017 (3)	0.018 (4)	−0.001 (3)	−0.001 (3)	0.007 (3)
O17	0.006 (3)	0.013 (3)	0.016 (4)	0.000 (2)	−0.003 (3)	0.008 (3)
O18	0.006 (3)	0.017 (3)	0.015 (3)	−0.001 (3)	−0.001 (3)	0.005 (3)
O19	0.016 (3)	0.009 (3)	0.016 (4)	0.001 (3)	−0.004 (3)	0.002 (3)
O20	0.021 (4)	0.008 (3)	0.018 (4)	0.005 (3)	−0.005 (3)	0.001 (3)
O21	0.013 (3)	0.011 (3)	0.016 (4)	0.002 (3)	0.001 (3)	0.008 (3)
O22	0.015 (3)	0.020 (3)	0.012 (4)	−0.003 (3)	−0.002 (3)	0.005 (3)
O23	0.007 (3)	0.011 (3)	0.016 (3)	−0.001 (2)	−0.001 (3)	0.007 (3)
O24	0.010 (3)	0.004 (3)	0.015 (3)	0.002 (2)	−0.003 (3)	−0.003 (3)
O25	0.019 (4)	0.027 (4)	0.026 (4)	−0.003 (3)	0.003 (3)	0.023 (3)
O26	0.012 (3)	0.023 (4)	0.024 (4)	0.009 (3)	0.001 (3)	0.004 (3)
O27	0.069 (6)	0.010 (4)	0.026 (5)	−0.012 (4)	−0.016 (4)	0.007 (3)

### Diytterbium enneaoxidotritellurate(IV) Geometric parameters (Å, º)

**Table:**

Yb1—O3	2.217 (6)	Yb6—O25^viii^	2.276 (7)
Yb1—O22^i^	2.256 (7)	Yb6—O21	2.295 (6)
Yb1—O14^i^	2.273 (6)	Yb6—O17^ix^	2.305 (6)
Yb1—O15^i^	2.374 (6)	Yb6—O10	2.531 (7)
Yb1—O12	2.386 (6)	Te1—O1	1.899 (6)
Yb1—O15^ii^	2.394 (6)	Te1—O2^ii^	1.906 (6)
Yb1—O14	2.437 (6)	Te1—O3	1.955 (6)
Yb1—O11^i^	2.485 (7)	Te1—O8	2.384 (6)
Yb2—O4^iii^	2.219 (6)	Te2—O4	1.886 (6)
Yb2—O7^iv^	2.249 (6)	Te2—O6	1.915 (6)
Yb2—O4^i^	2.271 (6)	Te2—O5	1.919 (6)
Yb2—O1^iv^	2.279 (6)	Te2—O26	2.526 (7)
Yb2—O2	2.367 (6)	Te3—O7	1.867 (6)
Yb2—O6^i^	2.374 (6)	Te3—O9	1.881 (6)
Yb2—O13	2.384 (6)	Te3—O8^x^	1.894 (6)
Yb2—O1^v^	2.737 (6)	Te3—O20	2.697 (7)
Yb3—O19	2.194 (6)	Te4—O10	1.868 (6)
Yb3—O2	2.223 (6)	Te4—O11	1.884 (6)
Yb3—O11	2.225 (6)	Te4—O12	1.926 (6)
Yb3—O16	2.227 (7)	Te4—O23	2.531 (6)
Yb3—O15	2.309 (6)	Te5—O13^i^	1.858 (6)
Yb3—O12^v^	2.425 (6)	Te5—O14	1.902 (6)
Yb3—O13	2.662 (6)	Te5—O15^i^	1.924 (6)
Yb3—O22	2.979 (7)	Te5—O5	2.691 (6)
Yb4—O5^i^	2.193 (6)	Te6—O16	1.855 (7)
Yb4—O6^vi^	2.215 (6)	Te6—O17^ix^	1.893 (6)
Yb4—O24	2.250 (6)	Te6—O18^v^	1.926 (6)
Yb4—O3	2.271 (6)	Te6—O21	2.380 (6)
Yb4—O7	2.292 (6)	Te7—O19	1.862 (7)
Yb4—O1	2.399 (6)	Te7—O20^iv^	1.884 (6)
Yb4—O9	2.476 (7)	Te7—O21	1.888 (6)
Yb5—O9	2.130 (6)	Te7—O10	2.645 (6)
Yb5—O27^vii^	2.176 (7)	Te8—O22	1.844 (7)
Yb5—O18^vii^	2.247 (7)	Te8—O23	1.868 (6)
Yb5—O26^vi^	2.275 (7)	Te8—O24^i^	1.895 (6)
Yb5—O17^vi^	2.396 (6)	Te8—O16	2.729 (7)
Yb5—O24	2.407 (7)	Te9—O25^i^	1.855 (6)
Yb5—O25	2.578 (7)	Te9—O26	1.859 (6)
Yb5—O27^vi^	3.014 (9)	Te9—O27	1.874 (8)
Yb6—O18	2.250 (6)	Te9—O5	2.974 (7)
Yb6—O23	2.253 (6)		
			
O3—Yb1—O22^i^	84.4 (2)	O25^viii^—Yb6—O21	149.5 (3)
O3—Yb1—O14^i^	78.6 (2)	O20^viii^—Yb6—O17^ix^	94.9 (2)
O22^i^—Yb1—O14^i^	110.2 (2)	O18—Yb6—O17^ix^	158.1 (2)
O3—Yb1—O15^i^	148.3 (2)	O23—Yb6—O17^ix^	81.6 (2)
O22^i^—Yb1—O15^i^	83.6 (2)	O25^viii^—Yb6—O17^ix^	89.4 (2)
O14^i^—Yb1—O15^i^	133.1 (2)	O21—Yb6—O17^ix^	68.8 (2)
O3—Yb1—O12	74.2 (2)	O20^viii^—Yb6—O10	110.5 (2)
O22^i^—Yb1—O12	140.0 (2)	O18—Yb6—O10	70.0 (2)
O14^i^—Yb1—O12	98.3 (2)	O23—Yb6—O10	72.4 (2)
O15^i^—Yb1—O12	97.2 (2)	O25^viii^—Yb6—O10	137.8 (2)
O3—Yb1—O15^ii^	80.7 (2)	O21—Yb6—O10	72.1 (2)
O22^i^—Yb1—O15^ii^	73.5 (2)	O17^ix^—Yb6—O10	126.1 (2)
O14^i^—Yb1—O15^ii^	158.4 (2)	O1—Te1—O2^ii^	89.2 (3)
O15^i^—Yb1—O15^ii^	67.8 (2)	O1—Te1—O3	83.8 (3)
O12—Yb1—O15^ii^	70.0 (2)	O2^ii^—Te1—O3	92.8 (3)
O3—Yb1—O14	136.8 (2)	O1—Te1—O8	86.4 (2)
O22^i^—Yb1—O14	136.9 (2)	O2^ii^—Te1—O8	78.6 (2)
O14^i^—Yb1—O14	74.9 (2)	O3—Te1—O8	167.0 (2)
O15^i^—Yb1—O14	66.2 (2)	O4—Te2—O6	84.7 (3)
O12—Yb1—O14	76.5 (2)	O4—Te2—O5	94.4 (3)
O15^ii^—Yb1—O14	117.7 (2)	O6—Te2—O5	93.5 (3)
O3—Yb1—O11^i^	130.1 (2)	O4—Te2—O26	163.3 (3)
O22^i^—Yb1—O11^i^	69.1 (2)	O6—Te2—O26	82.0 (2)
O14^i^—Yb1—O11^i^	72.7 (2)	O5—Te2—O26	76.5 (3)
O15^i^—Yb1—O11^i^	71.3 (2)	O7—Te3—O9	84.1 (3)
O12—Yb1—O11^i^	148.8 (2)	O7—Te3—O8^x^	99.8 (3)
O15^ii^—Yb1—O11^i^	126.6 (2)	O9—Te3—O8^x^	92.1 (3)
O14—Yb1—O11^i^	72.3 (2)	O7—Te3—O20	89.4 (3)
O4^iii^—Yb2—O7^iv^	71.8 (2)	O9—Te3—O20	86.1 (2)
O4^iii^—Yb2—O4^i^	65.0 (3)	O8^x^—Te3—O20	170.4 (3)
O7^iv^—Yb2—O4^i^	128.6 (2)	O10—Te4—O11	98.4 (3)
O4^iii^—Yb2—O1^iv^	90.8 (2)	O10—Te4—O12	103.3 (3)
O7^iv^—Yb2—O1^iv^	71.9 (2)	O11—Te4—O12	92.1 (3)
O4^i^—Yb2—O1^iv^	82.0 (2)	O10—Te4—O23	78.7 (2)
O4^iii^—Yb2—O2	123.4 (2)	O11—Te4—O23	83.2 (2)
O7^iv^—Yb2—O2	71.7 (2)	O12—Te4—O23	175.2 (2)
O4^i^—Yb2—O2	157.9 (2)	O13^i^—Te5—O14	103.0 (3)
O1^iv^—Yb2—O2	116.4 (2)	O13^i^—Te5—O15^i^	89.7 (3)
O4^iii^—Yb2—O6^i^	129.3 (2)	O14—Te5—O15^i^	86.8 (3)
O7^iv^—Yb2—O6^i^	157.4 (2)	O13^i^—Te5—O5	82.2 (2)
O4^i^—Yb2—O6^i^	66.8 (2)	O14—Te5—O5	99.3 (2)
O1^iv^—Yb2—O6^i^	97.3 (2)	O15^i^—Te5—O5	170.8 (2)
O2—Yb2—O6^i^	97.2 (2)	O16—Te6—O17^ix^	99.2 (3)
O4^iii^—Yb2—O13	90.2 (2)	O16—Te6—O18^v^	97.4 (3)
O7^iv^—Yb2—O13	116.7 (2)	O17^ix^—Te6—O18^v^	84.1 (3)
O4^i^—Yb2—O13	90.5 (2)	O16—Te6—O21	87.5 (3)
O1^iv^—Yb2—O13	171.2 (2)	O17^ix^—Te6—O21	73.9 (2)
O2—Yb2—O13	70.0 (2)	O18^v^—Te6—O21	157.9 (2)
O6^i^—Yb2—O13	75.4 (2)	O19—Te7—O20^iv^	95.8 (3)
O4^iii^—Yb2—O1^v^	154.0 (2)	O19—Te7—O21	96.6 (3)
O7^iv^—Yb2—O1^v^	88.8 (2)	O20^iv^—Te7—O21	96.9 (3)
O4^i^—Yb2—O1^v^	120.2 (2)	O19—Te7—O10	85.2 (2)
O1^iv^—Yb2—O1^v^	66.3 (2)	O20^iv^—Te7—O10	172.7 (3)
O2—Yb2—O1^v^	62.7 (2)	O21—Te7—O10	75.8 (2)
O6^i^—Yb2—O1^v^	68.62 (19)	O22—Te8—O23	102.6 (3)
O13—Yb2—O1^v^	114.33 (19)	O22—Te8—O24^i^	97.2 (3)
O19—Yb3—O2	74.8 (2)	O23—Te8—O24^i^	97.7 (3)
O19—Yb3—O11	93.8 (2)	O22—Te8—O16	75.0 (3)
O2—Yb3—O11	144.5 (2)	O23—Te8—O16	80.9 (2)
O19—Yb3—O16	88.9 (2)	O24^i^—Te8—O16	171.4 (2)
O2—Yb3—O16	113.1 (2)	O25^i^—Te9—O26	101.5 (3)
O11—Yb3—O16	99.8 (2)	O25^i^—Te9—O27	98.2 (3)
O19—Yb3—O15	141.9 (2)	O26—Te9—O27	89.3 (4)
O2—Yb3—O15	91.1 (2)	O25^i^—Te9—O5	85.2 (3)
O11—Yb3—O15	77.4 (2)	O26—Te9—O5	66.0 (3)
O16—Yb3—O15	128.9 (2)	O27—Te9—O5	155.2 (3)
O19—Yb3—O12^v^	132.2 (2)	Te1—O1—Yb2^iv^	133.7 (3)
O2—Yb3—O12^v^	70.3 (2)	Te1—O1—Yb4	102.2 (2)
O11—Yb3—O12^v^	133.1 (2)	Yb2^iv^—O1—Yb4	106.4 (2)
O16—Yb3—O12^v^	76.2 (2)	Te1—O1—Yb2^ii^	97.2 (2)
O15—Yb3—O12^v^	70.7 (2)	Yb2^iv^—O1—Yb2^ii^	113.7 (2)
O19—Yb3—O13	77.5 (2)	Yb4—O1—Yb2^ii^	98.0 (2)
O2—Yb3—O13	67.1 (2)	Te1^v^—O2—Yb3	124.1 (3)
O11—Yb3—O13	77.7 (2)	Te1^v^—O2—Yb2	110.6 (3)
O16—Yb3—O13	165.9 (2)	Yb3—O2—Yb2	116.5 (3)
O15—Yb3—O13	64.5 (2)	Te1—O3—Yb1	116.8 (3)
O12^v^—Yb3—O13	115.8 (2)	Te1—O3—Yb4	105.0 (2)
O19—Yb3—O22	136.8 (2)	Yb1—O3—Yb4	137.7 (3)
O2—Yb3—O22	146.0 (2)	Te2—O4—Yb2^xi^	134.0 (3)
O11—Yb3—O22	60.4 (2)	Te2—O4—Yb2^i^	106.5 (3)
O16—Yb3—O22	65.1 (2)	Yb2^xi^—O4—Yb2^i^	115.0 (3)
O15—Yb3—O22	70.1 (2)	Te2—O5—Yb4^i^	122.0 (3)
O12^v^—Yb3—O22	76.71 (19)	Te2—O6—Yb4^vi^	130.6 (3)
O13—Yb3—O22	123.25 (18)	Te2—O6—Yb2^i^	101.7 (2)
O5^i^—Yb4—O6^vi^	171.8 (2)	Yb4^vi^—O6—Yb2^i^	115.5 (3)
O5^i^—Yb4—O24	87.4 (2)	Te3—O7—Yb2^iv^	126.1 (3)
O6^vi^—Yb4—O24	98.7 (2)	Te3—O7—Yb4	107.8 (3)
O5^i^—Yb4—O3	87.4 (2)	Yb2^iv^—O7—Yb4	111.1 (3)
O6^vi^—Yb4—O3	86.8 (2)	Te3^x^—O8—Te1	111.5 (3)
O24—Yb4—O3	94.5 (2)	Te3^x^—O8—Te2^iii^	118.0 (3)
O5^i^—Yb4—O7	81.1 (2)	Te1—O8—Te2^iii^	123.5 (3)
O6^vi^—Yb4—O7	98.8 (2)	Te3—O9—Yb5	152.1 (4)
O24—Yb4—O7	129.9 (2)	Te3—O9—Yb4	100.4 (3)
O3—Yb4—O7	133.0 (2)	Yb5—O9—Yb4	104.7 (2)
O5^i^—Yb4—O1	94.8 (2)	Te4—O10—Yb6	106.7 (3)
O6^vi^—Yb4—O1	77.7 (2)	Te4—O11—Yb3	138.5 (3)
O24—Yb4—O1	161.1 (2)	Te4—O11—Yb1^i^	117.9 (3)
O3—Yb4—O1	66.9 (2)	Yb3—O11—Yb1^i^	98.0 (2)
O7—Yb4—O1	69.0 (2)	Te4—O12—Yb1	117.8 (3)
O5^i^—Yb4—O9	107.4 (2)	Te4—O12—Yb3^ii^	123.1 (3)
O6^vi^—Yb4—O9	79.6 (2)	Yb1—O12—Yb3^ii^	107.7 (2)
O24—Yb4—O9	74.2 (2)	Te5^i^—O13—Yb2	120.9 (3)
O3—Yb4—O9	160.6 (2)	Te5^i^—O13—Yb3	97.3 (2)
O7—Yb4—O9	63.4 (2)	Yb2—O13—Yb3	101.2 (2)
O1—Yb4—O9	122.5 (2)	Te5—O14—Yb1^i^	145.5 (3)
O9—Yb5—O27^vii^	152.5 (3)	Te5—O14—Yb1	102.4 (3)
O9—Yb5—O18^vii^	91.7 (2)	Yb1^i^—O14—Yb1	105.1 (2)
O27^vii^—Yb5—O18^vii^	95.0 (3)	Te5^i^—O15—Yb3	108.2 (3)
O9—Yb5—O26^vi^	79.5 (3)	Te5^i^—O15—Yb1^i^	104.0 (2)
O27^vii^—Yb5—O26^vi^	108.5 (3)	Yb3—O15—Yb1^i^	98.9 (2)
O18^vii^—Yb5—O26^vi^	144.8 (2)	Te5^i^—O15—Yb1^v^	119.9 (3)
O9—Yb5—O17^vi^	87.2 (2)	Yb3—O15—Yb1^v^	111.4 (2)
O27^vii^—Yb5—O17^vi^	119.9 (3)	Yb1^i^—O15—Yb1^v^	112.2 (2)
O18^vii^—Yb5—O17^vi^	66.7 (2)	Te6—O16—Yb3	140.9 (3)
O26^vi^—Yb5—O17^vi^	78.7 (2)	Te6^ix^—O17—Yb6^ix^	113.4 (3)
O9—Yb5—O24	77.8 (2)	Te6^ix^—O17—Yb5^vi^	101.2 (2)
O27^vii^—Yb5—O24	79.5 (3)	Yb6^ix^—O17—Yb5^vi^	144.8 (3)
O18^vii^—Yb5—O24	138.2 (2)	Te6^ii^—O18—Yb5^viii^	105.5 (3)
O26^vi^—Yb5—O24	73.5 (2)	Te6^ii^—O18—Yb6	123.6 (3)
O17^vi^—Yb5—O24	150.3 (2)	Yb5^viii^—O18—Yb6	117.8 (3)
O9—Yb5—O25	80.8 (2)	Te7—O19—Yb3	141.9 (4)
O27^vii^—Yb5—O25	78.0 (3)	Te7^iv^—O20—Yb6^vii^	124.2 (3)
O18^vii^—Yb5—O25	65.1 (2)	Te7—O21—Yb6	118.8 (3)
O26^vi^—Yb5—O25	144.2 (2)	Te7—O21—Te6	131.9 (3)
O17^vi^—Yb5—O25	129.7 (2)	Yb6—O21—Te6	97.5 (2)
O24—Yb5—O25	73.3 (2)	Te8—O22—Yb1^i^	142.4 (4)
O9—Yb5—O27^vi^	134.4 (2)	Te8—O22—Yb3	106.6 (3)
O27^vii^—Yb5—O27^vi^	65.5 (4)	Yb1^i^—O22—Yb3	84.5 (2)
O18^vii^—Yb5—O27^vi^	114.1 (2)	Te8—O23—Yb6	130.8 (3)
O26^vi^—Yb5—O27^vi^	57.5 (2)	Te8^i^—O24—Yb4	138.4 (3)
O17^vi^—Yb5—O27^vi^	71.4 (2)	Te8^i^—O24—Yb5	106.4 (3)
O24—Yb5—O27^vi^	101.3 (2)	Yb4—O24—Yb5	103.2 (2)
O25—Yb5—O27^vi^	143.4 (2)	Te9^i^—O25—Yb6^vii^	130.6 (4)
O20^viii^—Yb6—O18	91.6 (2)	Te9^i^—O25—Yb5	121.7 (3)
O20^viii^—Yb6—O23	176.4 (2)	Yb6^vii^—O25—Yb5	104.8 (2)
O18—Yb6—O23	91.4 (2)	Te9—O26—Yb5^vi^	120.9 (4)
O20^viii^—Yb6—O25^viii^	84.6 (3)	Te9—O26—Te2	111.1 (3)
O18—Yb6—O25^viii^	70.4 (2)	Yb5^vi^—O26—Te2	119.2 (3)
O23—Yb6—O25^viii^	94.5 (2)	Te9—O27—Yb5^viii^	131.9 (4)
O20^viii^—Yb6—O21	76.7 (2)	Te9—O27—Yb5^vi^	91.9 (3)
O18—Yb6—O21	133.1 (2)	Yb5^viii^—O27—Yb5^vi^	114.5 (4)
O23—Yb6—O21	102.7 (2)		

Symmetry codes: (i) −*x*+1, −*y*+1, −*z*+1; (ii) *x*−1, *y*, *z*; (iii) *x*, *y*−1, *z*; (iv) −*x*+1, −*y*, −*z*+1; (v) *x*+1, *y*, *z*; (vi) −*x*, −*y*+1, −*z*+1; (vii) *x*, *y*, *z*+1; (viii) *x*, *y*, *z*−1; (ix) −*x*+1, −*y*+1, −*z*; (x) −*x*, −*y*, −*z*+1; (xi) *x*, *y*+1, *z*.
